# The Use of Artificial Intelligence in Predicting Chemotherapy-Induced Toxicities in Metastatic Colorectal Cancer: A Data-Driven Approach for Personalized Oncology

**DOI:** 10.3390/diagnostics14182074

**Published:** 2024-09-19

**Authors:** Eliza-Maria Froicu, Oriana-Maria Oniciuc, Vlad-Adrian Afrăsânie, Mihai-Vasile Marinca, Silvia Riondino, Elena Adriana Dumitrescu, Teodora Alexa-Stratulat, Iulian Radu, Lucian Miron, Gema Bacoanu, Vladimir Poroch, Bogdan Gafton

**Affiliations:** 1 Department of Medical Oncology, Regional Institute of Oncology, 700483 Iasi, Romania; eliza-maria.froicu@umfiasi.ro (E.-M.F.);; 2 Department of Oncology, Faculty of Medicine, “Grigore T. Popa” University of Medicine and Pharmacy, 700115 Iasi, Romania; 3 2nd Internal Medicine Department, Faculty of Medicine, “Grigore T. Popa“ University of Medicine and Pharmacy, 700115 Iasi, Romania; 4 Faculty of Computer Science, “Alexandru Ioan Cuza” University, 700506 Iasi, Romania; 5 Department of Systems Medicine, Medical Oncology, Tor Vergata Clinical Center, University of Rome “Tor Vergata”, Viale Oxford 81, 00133 Rome, Italy; 6 Department of Oncology, Faculty of Medicine, “Carol Davila” University of Medicine and Pharmacy, 050474 Bucharest, Romania; 7 Institute of Oncology Prof. Dr. Alexandru Trestioreanu, Șoseaua Fundeni, 022328 Bucharest, Romania; 8 First Surgical Oncology Unit, Department of Surgery, Regional Institute of Oncology, 700483 Iasi, Romania; 9 Department of Surgery, Faculty of Medicine, “Grigore T. Popa” University of Medicine and Pharmacy, 700115 Iasi, Romania; 10 Department of Palliative Care, Regional Institute of Oncology, 700483 Iasi, Romania

**Keywords:** metastatic colorectal cancer, artificial intelligence, prediction model, chemotherapy toxicity

## Abstract

Background: Machine learning models learn about general behavior from data by finding the relationships between features. Our purpose was to develop a predictive model to identify and predict which subset of colorectal cancer patients are more likely to experience chemotherapy-induced toxicity and to determine the specific attributes that influence the presence of treatment-related side effects. Methods: The predictor was general toxicity, and for the construction of our data training, we selected 95 characteristics that represent the health state of 74 patients prior to their first round of chemotherapy. After the data were processed, Random Forest models were trained to offer an optimal balance between accuracy and interpretability. Results: We constructed a machine learning predictor with an emphasis on assessing the importance of numerical and categorical variables in relation to toxicity. Conclusions: The incorporation of artificial intelligence in personalizing colorectal cancer management by anticipating and overseeing toxicities more effectively illustrates a pivotal shift towards more personalized and precise medical care.

## 1. Introduction

Colorectal cancer (CRC) has become one of the most important cancers world-wide. Current guidelines recommend chemotherapy as adjuvant therapy for some stage II and most stage III cancers and as a backbone systemic treatment for stage IV cases. In either case, it may have a huge impact on progression-free survival and overall survival, but this often must be weighed against the downside of adverse events. Notably, two types of monoclonal antibodies that work against the epidermal growth factor receptor (anti-EGFR) and the vascular endothelial growth factor (anti-VEGF), respectively, have become game changers after being approved for use in a first-line setting for metastatic CRC (mCRC) treatment [1]. The median overall survival of patients with mCRC has exceeded 30 months with these novel treatments [2]; however, targeted therapies have unique toxicity profiles, including rash, diarrhea, hypertension, hypothyroidism, proteinuria, depigmentation, and hepatotoxicity. Research has demonstrated a correlation between these toxicities and therapeutic responses [3].

In clinical practice, more than 21% of CRC patients receive adjuvant chemotherapy for stage II tumors [4], as do 60% of stage III patients, and almost all stage IV patients with good performance status [5]. The need to forecast adverse outcomes in this large cohort (especially during the first chemotherapy cycle) thus validates, in our opinion, the rationale of the current experiment, which builds upon other available tools such as “ColonPrediscores” (adapted for senior patients) that take into consideration factors like polychemotherapy, hypoalbuminemia, C-reactive protein, ECOG PS, metastatic disease, age, alkaline phosphatase, and sex. These factors were considered independent predictors in the development of our model [6].

The incorporation of artificial intelligence (AI) into the management and treatment of colorectal cancer illustrates a pivotal shift towards more personalized and precise medical care. As highlighted by Yang et al., AI’s utility spans across various critical aspects, from selecting treatment strategies to evaluating patient prognoses, marking significant advancements in the field [7].

For instance, the prediction of patient survival has been enhanced through AI models that analyze histological images to identify microenvironment biomarkers. This approach enables a more nuanced understanding of the cancer’s behavior and potential outcomes, tailoring prognosis evaluations with a higher degree of accuracy [8]. Similarly, in the realm of metastasis prediction, the application of machine learning algorithms to predict liver metastases in patients with early-stage (T1) colorectal cancer at the initial diagnosis demonstrates AI’s capacity to identify patients at higher risk. This early detection is crucial for planning more effective treatment strategies, potentially improving overall survival rates [9]. Furthermore, AI’s role extends beyond diagnostics and prognostics to direct therapeutic decision making. By integrating vast datasets and uncovering patterns that may not be immediately apparent to humans, AI systems can recommend treatment plans that are optimized for individual patient profiles. This includes identifying which patients are more likely to benefit from specific chemotherapy regimens, targeted therapies, or surgical interventions, thereby enhancing the efficacy of treatments.

In the past decade, there have been efforts in the implementation of machine learning (ML) and artificial intelligence (AI) for the prediction of complications associated with the systemic therapy of cancer [10,11,12]. By analyzing datasets and incorporating multiple variables, ML and AI can provide valuable insights to healthcare providers, enabling them to customize treatment plans and minimize the occurrence of severe toxicities. Furthermore, AI-driven models can constantly gain knowledge from real-world feedback to boost their signature performance. To implement these methods, the FDA has proposed a regulatory framework for deploying AI-based technology as medical devices [13].

Building on the momentum of utilizing AI in colorectal cancer management, our study aimed to leverage diverse modeling techniques to investigate whether genetic backgrounds or other patient characteristics could predict treatment-related toxicity. This exploration is critical for enhancing the management of adverse effects stemming from cytotoxic therapies. Echoing the advancements in AI applications, such as survival and metastasis prediction, we hypothesized that patients’ characteristics play a significant role in an individual’s susceptibility to the adverse outcomes of cancer treatments. Our goal was to integrate this understanding into the broader context of AI’s impact on colorectal cancer treatment strategies that account for genetic predispositions and patient-specific factors.

## 2. Materials and Methods

### 2.1. Study Endpoint

The primary objective of the study was to develop a machine-learning model capable of identifying key features relevant to predicting toxicity following the initial chemotherapy treatment in colorectal cancer patients. This attempt sought to transcend the context of the treatment setting, whether adjuvant or metastatic, to ensure broad applicability and support in managing and mitigating the adverse effects associated with chemotherapy regimens.

### 2.2. Patient Data Preprocessing

**Figure 1:**
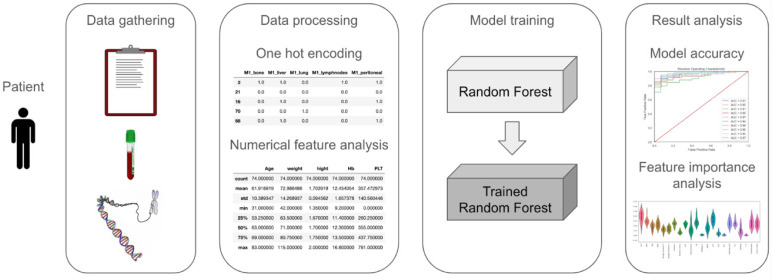
A graphic representation of the data processing framework’s structure.

The dataset contains entries for 74 patients, with a total of 95 initial features. We split the attributes by their type into categorical and numerical features and transformed the categorical features through one-hot encoding. This resulted in a total of 140 features. Toxicity appeared in 57 patients (77.0%). We defined any patient who experienced at least one type of adverse effect as having encountered toxicity. Recorded toxicities were transformed into binary form (yes/no), and we considered that toxicity was present (yes) if at least a grade 1 event was documented. The predictor was general toxicity, and for training, we selected 95 properties that represent the patient’s health state before the first round of chemotherapy (Figure 1).

### 2.3. Study Population

Our research included data from 74 colorectal cancer patients who were treated consecutively between January 2018 and December 2019 at the Regional Institute of Oncology in Iași, Romania, provided they complied with the inclusion and exclusion criteria listed below.

#### 2.3.1. Main Inclusion Criteria

The main inclusion criteria are as follows:

- Age over 18 years old;
- ECOG status of 0, 1, or 2;
- Pathologically confirmed colon or rectal adenocarcinoma;
- TNM stage II, III, or IV;
- Tissue sample available for genetic testing;
- Chemotherapy administration of first intent, at least one cycle;
- Evaluation of toxicity after the first cycle of chemotherapy, including late side-effects;
- Adequate bone marrow function, in the opinion of the investigator, or acceptable bone marrow function necessary to comply with the local protocol of chemotherapy administration.

#### 2.3.2. Main Exclusion Criteria

The main exclusion criteria are as follows:

- Prior chemotherapy for colorectal cancer or other cancers;
- Active infection, including tuberculosis, hepatitis B, hepatitis C, and HIV;
- Loss of follow-up and inability to monitor adverse events after chemotherapy.

### 2.4. Treatment Regimens and Associated Toxicity

Patients were enrolled in our trial only if they were subjected to chemotherapy, meaning that they were administered at least one cycle of chemotherapy and the toxicity was evaluated after. Patients may have also received biologic treatment (in concordance with the mutational status of all-RAS and BRAF) for metastatic disease. According to international guidelines, some patients received monotherapy (capecitabine or 5-fluorouracil) or doublet treatment (oxaliplatin or irinotecan plus fluoropyrimidine backbone). At the start of the trial, immunotherapy was not a standard first-line treatment for metastatic disease. As a result, testing of MSI or MMR status was not mandatory; when available, however, these data were included in our analysis.

After the first cycle of chemotherapy, a thorough set of serum chemistry, hematology, and coagulation tests were performed and then analyzed for the aim of the research. The examinations were conducted at either 14 or at 21 days following chemotherapy, depending on the regimen protocol. Treatment effectiveness assessment was not an endpoint in this trial and was not mandatory, as our main focus was on evaluating safety. National Cancer Institute—Common Toxicity Criteria version 5.0 was used to assess toxicity [14].

### 2.5. Machine Learning Models

We modeled the problem as a categorical predictor. For handling the large number of features compared to the total patients in the study, we chose random forest (RF) models. For creating the Random Forest model, the dataset is split into sub-datasets. Each sub-dataset is used to construct a decision tree (DT), in the end forming a forest of many trees. The strength of RF lies in its assembly of multiple DTs, enabling it to achieve higher and more robust performance by better generalizing across the dataset.

Another characteristic of RF that is useful for our case is that, for each tree trained in each iteration, the model analyzes a relatively small number of features (we used a maximum of the square root of a total number of features). We aim through this approach to overcome the “curse of dimensionality” induced by the number of features being larger than the number of patients. For predicting a new input sample, each DT in the RF will classify the sample and output a classification result which will be counted as one vote to the entire forest. The classification result will be the class with the highest score.

In the model training flow, we first created a dataset consisting of 95 features and one prediction column representing true if any type of toxicity was present and one representing false for no toxicity reported. Due to the difference in the number of patients with toxicity and without, we set the class weight to “balanced”. The max depth of the trees trained is 2, and we generated 100 trees per model.

### 2.6. Statistical Analysis

Statistical analysis was performed using SPSS v25.0 (SPSS, Inc, Chicago, IL, USA). Group comparisons were made using a Chi-square test, while quantitative and ordered variables were compared using the Mann–Whitney U test. For comparisons among three or more groups, a one-way ANOVA was used. A two-sided *p* value ≤ 0.05 was considered statistically significant. Python (version 3.10.9) was utilized to perform statistical analyses. The following Python packages were listed: ‘pandas’, ‘sklearn’, ‘seaborn’, and ‘matplotlib’. Demographic differences between the two subgroups were tested utilizing either Student’s *t*-test or Pearson chi-square test.

## 3. Results

### 3.1. Patients’ Characteristics

Our research was based on characteristics from 74 patients. In the study group, female patients were slightly more numerous than male patients (68% vs. 39%). The median age in the study group was 63 years, and the majority of patients were 65 years or older (53.8%). Liver metastases were the most frequent (59.6%), followed by peritoneal metastases (34.6%). In our study, the most used treatment in the first-line setting was Capecitabine + Oxaliplatin (CAPOX), followed by folinic acid, fluorouracil and oxaliplatin (FOLFOX), and Capecitabine monotherapy (Table 1).

### 3.2. Patients’ Toxicity Profile

**Figure 2:**
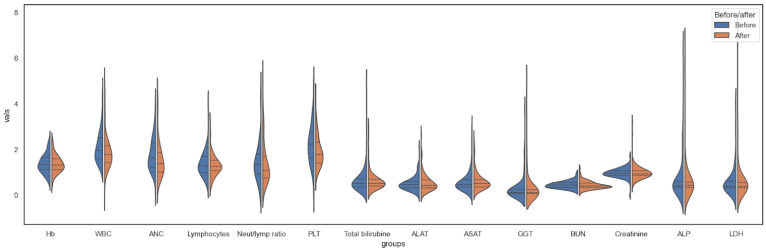
The distribution of the hematology and chemistry parameter groups before and after chemotherapy.

Following chemotherapy, a notable reduction was observed in the levels of white blood cells, neutrophil count, neutrophil-to-lymphocyte ratio, and platelets. Concurrently, there was a significant elevation in the levels of gamma-glutamyl transferase (GGT), creatinine, and lactate dehydrogenase (LDH), as seen in Figure 2 and Table 2.

### 3.3. Model Validation

**Figure 3:**
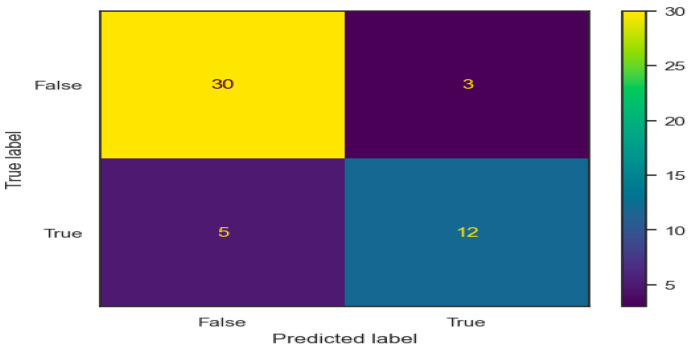
Confusion matrix for model validation.

**Figure 4:**
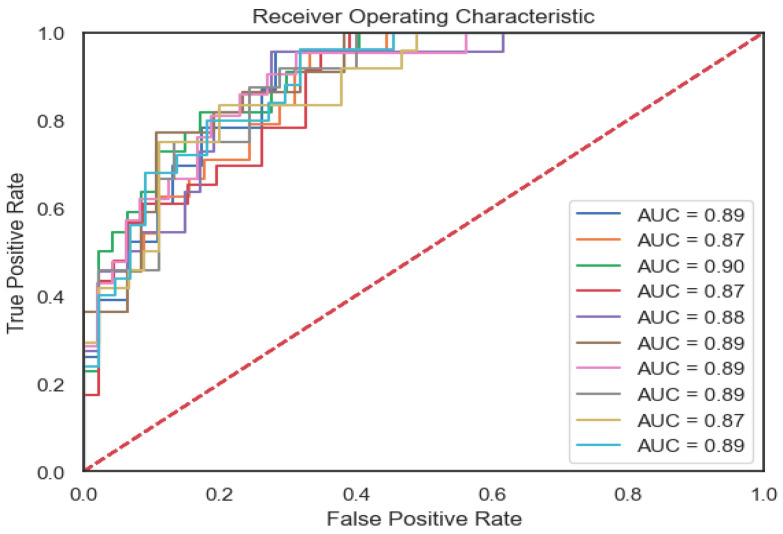
ROC curve for model validation.

We ran 10 independent experiments wherein we shuffled the dataset and split the train set and test set following a k-fold cross-validation procedure. The confusion matrix shows the imbalance in classes, but the model reaches a good prediction for the False (No Toxicity) class (Figure 3). The ROC curve presented (AUC ranging from 0.91 to 0.97 on the training set) proves that the model learned the relationships between features, justifying the possibility of using the model for real-world data prediction (Figure 4).

### 3.4. Important Categorical Variables

Curable disease, treatment setting (neoadjuvant, adjuvant, or metastatic), mucinous adenocarcinoma, T4b stage (tumor grown into or attached to other organs or structures), oligometastatic disease, absence of biologic treatment, M1c (cancer spread to distant parts of the peritoneum), history of smoking, and KRAS mutations are the most important categorical variables. The identified attributes are considered to have a significant influence on the model’s predictions, meaning that the presence or absence of these parameters greatly impacts the outcome the model predicts and will always increase or decrease the likelihood of a specific outcome in a linear way, i.e., a positive or negative correlation (Table 3).

### 3.5. Important Numerical Variables

**Figure 5:**
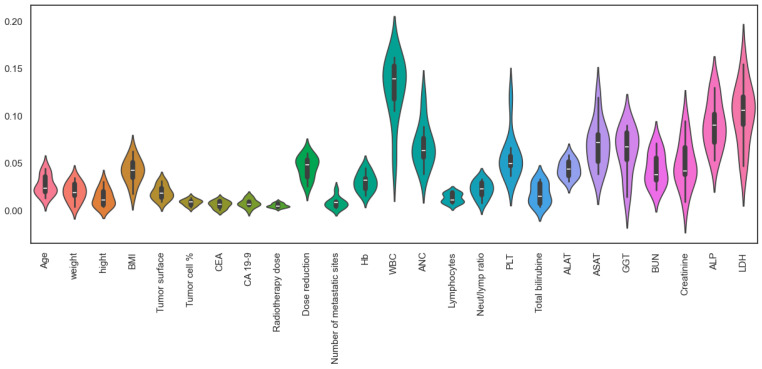
Numerical features framework.

White blood cells (WBCs), lactate dehydrogenase (LDH), alkaline phosphatase (ALP), aspartate aminotransferase (ASAT), absolute neutrophil count (ANC), gamma-glutamyl transferase (GGT), platelets, creatinine, dose reduction, and blood urea nitrogen are identified as the most important numerical variables for our model (Figure 5, Table 4).

## 4. Discussion

Over time, researchers have been diligently working to identify factors that can predict treatment toxicity. For example, one prospective cohort study aimed to develop and validate a risk model for neutropenic complications in 3760 patients with common solid tumors or malignant lymphoma starting a new chemotherapy regimen across 115 practice sites in the United States. A regression model was developed and validated using a random split-sample selection process. Results showed no significant differences between the derivation and validation populations. Neutropenic complications were most pronounced in the first cycle, with major independent risk factors including prior chemotherapy, abnormal hepatic and renal function, low white blood cell count, and chemotherapy dose delivery ≥85%. The model demonstrated good performance, with a sensitivity of 90%, specificity of 59%, and positive and negative predictive values of 34% and 96%, respectively, at a predicted risk cut point of 10%. Further analysis confirmed the model’s ability to discriminate the risk of febrile neutropenia over multiple chemotherapy cycles, thus offering valuable guidance for the optimal and cost-effective use of supportive care in these patients [15].

Based on various references, several factors have been identified as potential predictors of chemotherapy-induced toxicity specifically in colorectal cancer patients. Wang et al. proposed a risk prediction nomogram for fluoropyrimidine-induced cardiotoxicity in colorectal cancer, providing a tool for patient counseling and risk stratification before starting chemotherapy [16]. Deenen et al. discussed the relationship between single nucleotide polymorphisms and haplotypes in the dihydropyrimidine dehydrogenase (DPD) gene, and the toxicity and efficacy of capecitabine in advanced colorectal cancer, indicating the potential of genetic biomarkers in predicting chemotherapy-related adverse events. Other clinical parameters have also been associated with chemotherapy-induced toxicity in colorectal cancer [17]. Yahagi et al. reported the predictive value of the non-alcoholic fatty liver disease fibrosis score in anticipating hematological toxicity of chemotherapy for colorectal cancer [18], while Park et al. addressed the challenge of chemotherapy-induced neurotoxicity, emphasizing the assessment strategies, neuroprotective approaches, and potential treatments [19].

While it is common practice for clinicians to expect adverse events after chemotherapy, there is a rising interest in using machine learning (ML) models to optimize cancer therapy. These models aim to enhance treatment efficacy and minimize toxicity on an individual patient basis. Wiberg et al. developed an ML-based neutropenia prediction model to assess the risk at the initiation of a chemotherapy cycle [20]. Cho et al. also employed ML to improve the prediction of febrile neutropenia in patients undergoing chemotherapy for breast cancer [21]. Furthermore, Cuplov and André discussed the use of an ML approach to forecast chemotherapy-induced hematological toxicities in patients with rhabdomyosarcoma [22]. These studies showcase the vast potential of machine learning for accurately predicting the toxicity of chemotherapy.

In our study, which focuses on predicting any type of drug-related toxicity in colorectal patients after the first administration of chemotherapy, we exclusively utilized the Random Forest algorithm. This approach contrasts with the work by Li Chao et al., who developed ML models specifically to forecast fluoropyrimidine-induced cardiotoxicity in colorectal cancer patients. In their research, the XGBoost algorithm emerged as the most precise, achieving a score of 0.607 [23]. Despite the difference in focus and algorithms used, both studies highlight the potential of ML models in accurately predicting chemotherapy-related toxicities. Our study, with its use of the Random Forest algorithm, contributes to the broader prediction of the global risk for chemotherapy toxicities, beyond cardiotoxicity.

Another research initiative focused on forecasting adverse reactions from eight chemotherapy treatments (FOLFOX, FOLFIRI, paclitaxel, and GP) using electronic health record (EHR) data and three distinct ML algorithms: logistic regression, decision trees, and artificial neural networks. They aimed to predict various side effects such as nausea, vomiting, fatigue, anorexia, diarrhea, peripheral neuropathy, hypersensitivity, stomatitis, hand–foot syndrome, and constipation. Out of these, eight adverse drug reactions were successfully predicted by the models, with logistic regression identified as the most effective method for these prediction tasks [24]. Since we found that Random Forest is also a valuable tool to that effect, this difference could suggest that the effectiveness of an ML algorithm can vary significantly depending on the context, including the types of toxicities being predicted, the dataset’s characteristics, and the specific treatment regimens involved.

Additional work has shown that ML models can forecast the toxicity of Irinotecan in each treatment cycle, specifically focusing on leukopenia, neutropenia, and diarrhea as the outcomes of interest. Random Forest (RF) was identified as the optimal technique for predicting leukopenia in this research, which further supports our option to choose the RF models for handling the large number of features compared to the total number of patients in the study [25]. This suggests that RF is not only effective across different settings and types of toxicity predictions but also across various chemotherapy regimens, in this case, Irinotecan. This consistency in algorithm performance strengthens the case for using Random Forest in predictive models for chemotherapy toxicities, highlighting its reliability and effectiveness in handling complex medical data and predicting adverse outcomes with significant clinical implications.

### 4.1. Important Categorical Variables and Clinical Relevance

#### 4.1.1. Curable Disease, Treatment Setting, Oligometastatic Disease

The factors that showed high scores in our study were curable disease, treatment setting (adjuvant or metastatic), and oligometastatic disease, with scores of 0.080979, 0.060790, and 0.031902, respectively. It is important to mention that the chemotherapy was administered in different settings; in metastatic disease, treatment was given with palliative intent, so dose reduction may occur frequently. For instance, Soveri et al. examined the relationship between adverse events and survival outcomes in colorectal cancer (CRC) patients undergoing treatment with adjuvant 5-fluorouracil (5FU) and leucovorin. Their investigation aimed to assess whether clinically assessable toxicities are linked with patient outcomes in early-stage CRC cases. The study findings underscore the predictive significance of adverse events such as neutropenia, mucositis, and nausea/vomiting in predicting survival outcomes, including disease-free survival (DFS) and overall survival (OS), in adjuvant CRC patients treated with 5FU [26]. Furthermore, as categorical variables, additional research is required to investigate if the association between the mentioned variables and the treatment-related toxicity is positive or negative and how this might be interpreted in clinical practice.

#### 4.1.2. Mucinous Adenocarcinoma

Mucinous adenocarcinoma is a distinct subtype of colorectal cancer and is characterized by abundant mucinous components that comprise at least 50% of the tumor volume [27]. Patients with mucinous colorectal adenocarcinoma currently receive treatments based on the same standard guidelines as for CRC. Liu et al. found that the FOLFIRI regimen could prolong PFS by 5 months compared with the FOLFOX-4 regimen for mucinous colorectal adenocarcinoma patients (*p* = 0.038), suggesting that the FOLFIRI regimen could be considered first for this group of patients [28]. Proximal tumors are more frequently mucinous, associated with an inflammatory response, with dMMR/MSI-H and hypermutated, with a higher frequency of KRAS and BRAF mutations. When dealing with a negative prognosis, it is common practice to administer the full dosage of medication, which might correlate with a more significant level of adverse events.

#### 4.1.3. T4a and M1c

T4 tumors have a greater risk of micrometastases. Consequently, they may require more intensive treatment regimens, including higher doses of chemotherapy. Results from the IDEA study suggested that patients with T4 colorectal cancers may benefit from maintaining a chemotherapy dose intensity (DI) of over 80%. This recommendation is based on the observed trend towards improved survival in this subgroup when DI was higher [29]. This increased intensity of treatment comes with a higher likelihood of experiencing adverse effects and toxicities. In our analysis, T4a achieved a score of 0.039078, indicating its significance as a robust clinical variable within the framework of toxicity prediction. Nie et al. developed a prognostic nomogram for estimating overall survival in locally advanced colorectal cancer (LACRC) patients, analyzing data from 636 individuals treated between 2017 and 2019. The study found TNM staging a significant predictor of survival [30]. The nomogram demonstrated superior ability in predicting patient outcomes, offering a valuable tool for personalized treatment planning. This implies a more complex approach to the TNM classification system, in which various stages not only impact survival results but also predict a higher risk of treatment-related problems. As with our study, both emphasize the need for precise TNM staging in personalizing treatment options, improving the capacity to predict patient outcomes, and managing the adverse effects of treatments such as chemotherapy in LACRC.

#### 4.1.4. Smoking

Substantial data are suggesting inter-individual variation in the pharmacokinetics of anticancer drugs are associated with clinically meaningful variability in toxicity and efficacy. For example, Jassem et al. undertook a comprehensive evaluation of the impact of tobacco smoke on the efficacy and tolerance of systemic therapy in cancer treatment. In their work, they discuss preclinical evidence suggesting nicotine may impair chemotherapy efficacy via a variety of cellular mechanisms, including the activation of growth and survival pathways, as well as its role in conferring apoptosis resistance. One other highlighted aspect is that smoking alters drug clearance and toxicity profiles, potentially necessitating tailored dosage adjustments for optimal therapeutic outcomes in smokers [31]. One meta-analysis by Bergman et al. analyzed the differential impact of smoking on cancer treatment outcomes and toxicity. When referring to smoking and chemotherapy-related toxicity, one analysis of nine studies, encompassing 3307 patients and examining data on 13 different toxicities from chemotherapy, revealed no statistically significant difference in the risk of chemotherapy-induced toxicity between smokers and non-smokers, with a pooled Odds Ratio (OR) of 0.92 and a 95% Confidence Interval (CI) ranging from 0.53 to 1.60. Furthermore, subgroup analyses were conducted for two specific types of chemotherapy, taxanes and platinum-based therapies, to ascertain if smoking influenced the risk of toxicity associated with these treatments. However, these analyses did not demonstrate any association between smoking and an increased risk of toxicity for either type of chemotherapy treatment [32]. Another study led by Peppone et al. [33] investigated the impact of cigarette smoking on symptom burden among 947 cancer patients undergoing treatment and at a 6-month follow-up. Smokers experienced significantly higher total symptom burdens compared to nonsmokers both during treatment and at the 6-month mark. However, participants who quit smoking before treatment showed symptom burdens similar to nonsmokers. The findings underscore the association between smoking and increased symptom burden during and after cancer treatment, emphasizing the importance of targeted cessation efforts to alleviate symptoms, potentially reducing treatment interruptions and enhancing post-treatment quality of life [33].

#### 4.1.5. KRAS

The presence of RAS mutations in colorectal cancer (CRC) has profound implications, as it impacts tumor progression, proliferation, and resistance to treatment. Therefore, it is imperative to determine the RAS mutation status in all stage IV CRC patients. Guo et al. demonstrated that KRAS mutations are linked to reduced overall survival (OS) in stage IV colorectal cancer (CRC) [34]. Another Chinese study on stage IV colorectal cancer patients found that the presence of KRAS mutation and the primary tumor location did not influence the efficacy of bevacizumab-containing chemotherapy. In addition, these characteristics were not shown to be predictive factors for survival outcomes in this cohort. However, the safety profiles or the impact of the existence of KRAS mutation were not thoroughly examined [35]. Several predictors included in our model have been previously identified as risk factors for toxic chemotherapy effects: WBC, LDH, ALP, ASAT, ANC, GGT, platelets, and creatinine. Huri et al. aimed to elucidate the risk factors associated with chemotherapy toxicity in the elderly patient population and to establish a risk stratification framework. They included pertinent variables, including geriatric assessment parameters, laboratory test results (e.g., creatinine clearance levels below 34 mL/min), and various patient, tumor, and treatment characteristics into a predictive model for grade 3 to 5 toxicity, seeking to provide clinicians with a valuable tool for assessing and mitigating the risk of chemotherapy toxicity in this vulnerable population [36]. Similarly, Extermann et al. enrolled patients aged 70 years and older initiating chemotherapy in their prospective, multicentric study. They constructed the Chemotherapy Risk Assessment Scale for High-Age Patients (CRASH) score, consisting of two subscores for hematologic (H) and nonhematologic (NH) toxicity. Predictors of H toxicity included lymphocytes, aspartate aminotransferase level, Instrumental Activities of Daily Living score, lactate dehydrogenase level, diastolic blood pressure, and Chemotox. Predictors of NH toxicity included hemoglobin and creatinine clearance [37]. The alignment between our results and the CRASH score highlights the robustness and generalizability of our findings within the broader context of chemotherapy toxicity prediction in older patients. Hyman et al. aimed to develop a nomogram for predicting the risk of serious drug-related toxicity (SDRT) in cycle one of phase I trials. Using data from Cancer Therapeutics Evaluation Program-sponsored trials, factors such as Eastern Cooperative Oncology Group performance status, white blood cell count, creatinine clearance, albumin, aspartate aminotransferase, number of study drugs, presence of a biologic study drug, and dose relative to the maximum administered were identified as significant predictors of SDRT. The final nomogram, excluding dose, was validated internally and externally, demonstrating its utility in accurately predicting a patient’s risk of SDRT at enrollment [38]. Lactate dehydrogenase (LDH) levels have been linked with the global tumor burden in several tumor types and can act as a tumor prognosis indicator and aid in evaluating the efficacy of systemic treatments. In the context of personalized cancer immunotherapy, Dercle et al. utilized AI algorithms, including Random Forest models, to determine which routine baseline medical parameters could most effectively predict resistance to anti-PD-L1 immunotherapies and reduced overall survival (OS) in cancer patients. Their findings highlighted that an elevation in serum lactate dehydrogenase was an independent predictor of limited OS in solid tumors [39]. Additionally, a meta-analysis indicated that higher LDH concentrations are associated with poorer overall survival rates in patients with metastatic colorectal cancer (mCRC) [40]. Despite this correlation, there is a noted deficit in research exploring the connection between elevated LDH levels and an increased risk of experiencing certain chemotherapy side effects or toxicities. In our study, LDH emerged as a very significant feature, offering clinicians a valuable tool to anticipate any form of toxicity, particularly neurological ones. This insight underscores the potential of LDH not only as a marker for cancer prognosis but also for its predictive value in anticipating chemotherapy-induced toxicities. Elevated levels of serum alkaline phosphatase (ALP) are an indicator of hepatobiliary damage. ALP levels are frequently increased in patients with colorectal cancer (CRC), especially in those with hepatic metastases [41]. Maisano et al. discussed the prognostic significance of ALP levels in patients with metastatic colorectal cancer undergoing FOLFOX 4 chemotherapy. In their dataset, patients with high alkaline phosphatase levels experienced significantly shorter median time to progression and overall survival compared to those with lower levels. Moreover, a difference in treatment response and toxicity profiles between the two groups was observed; in our research, ALP also emerged as a significant parameter for predicting chemotherapy toxicity risk [42].

### 4.2. Strengths and Limitations

One of the limitations of this study is that the number of patients was relatively small, and the number of parameters to analyze was large. Although the cohort was homogeneous, the collection of our parameters was gathered from real-world data rather than randomized controlled trials, reflecting the actual clinical aspects of the mCRC population in the eastern area of Romania. This study analyzed all types of toxicity together, and a better understanding of each patient would be provided through a different analysis per type of toxicity. Finally, although the models yield good metrics for the quality of the prediction, a new validation cohort would be helpful to assess the potential of our model as a predictor for chemotoxicity.

An important strength of this experiment is the training of an easily interpretable model able to predict chemotherapy toxicity. The variables used (blood test results and clinical measurements, as well as parameters deduced from discussion with the patient) are easily and consistently available, regardless of the clinical setting.

## 5. Conclusions

In our study, which focuses on predicting any type of drug-related toxicity in colorectal cancer patients after the first administration of chemotherapy, we did not specifically investigate the probable synergy between chemotherapy and biologic treatments in relation to side effects. Instead, our analysis centered on the patient’s characteristics that might predict different types of toxicity, regardless of the specific drug class administered. This approach allowed us to develop a more generalized model for predicting toxicity without delving into the intricate interactions between different drug classes. However, it is important to note that, for instance, cutaneous toxicity, particularly in the context of anti-EGFR therapy, has been studied as a prognostic marker of response to therapy. Given its significance, we recognize the value of exploring this association further. As such, we aim to consider the relationship between biologic treatment-induced cutaneous toxicity and overall treatment outcomes as a future research direction. This would help to better understand how the inclusion of biologic treatments influences the overall toxicity profile and treatment efficacy, thereby refining our predictive models and improving patient care strategies. Further expansion of this research could include different therapeutic regimens, as well as different neoplasms.

To bring our discussion to a close, our machine learning model is designed as a supportive tool to assist clinicians in identifying patients who possess certain features that may require closer follow-up due to an increased risk of treatment-related side effects. Our research is a proof of concept that illustrates the potential use of artificial intelligence in analyzing patients’ biological and tumoral characteristics to predict future adverse events, which, if not anticipated, could cause a delay in their subsequent chemotherapy administrations and compromise treatment results.

## Figures and Tables

**Table 1:** Patient’s characteristics.

Characteristics	Categories	No. of Patients (%)
Total Patients		74 (100%)
Median Age (years)		63
Sex	Male	35 (68%)
	Female	39 (37.5%)
Age	<65 years	48 (46.2%)
	≥65 years	56 (53.8%)
Smoking Status	Non-smokers	98 (94.2%)
	Smokers	6 (5.8%)
ECOG PS	0	5 (4.8%)
	1	89 (85.6%)
	2	10 (9.6%)
Primary Tumor Location	Left colon	82 (78.8%)
	Right colon	22 (21.1%)
Location of Metastases	Liver	62 (59.6%)
	Peritoneal	36 (34.6%)
	Lung	18 (17.4%)
	Other sites	9 (8.7%)
Histopathological Type	Ulcerated	84 (80.8%)
	Mucinous	16 (15.4%)
	Signet ring cell	4 (3.8%)
Grading	G1	8 (7.7%)
	G2	86 (82.7%)
	G3	10 (9.6%)
Primary Tumor Surgery	No	33 (31.7%)
	Yes	71 (68.3%)
Metastases Surgery	No	92 (88.5%)
	Yes	12 (11.5%)
First-Line Chemotherapy Regimen	FOLFOX	32 (30.8%)
	CAPOX	45 (43.3%)
	FOLFIRI	10 (9.6%)
	CAPEIRI	2 (1.9%)
	FUFOL	2 (1.9%)
	Capecitabine	13 (12.5%)
First-Line Biological Treatment	Bevacizumab	55 (52.9%)
	Cetuximab	16 (15.4%)
	Panitumumab	6 (5.8%)
	None	26 (25%)
KRAS Mutation	exon 2	42 (40.4%)
*n* = 47 (45.2%)	exon 3	3 (2.9%)
	exon 4	2 (1.9%)
NRAS Mutation	exon 2	1 (1%)
*n* = 5 (4.8%)	exon 3	4 (3.8%)
	exon 4	0 (0%)
BRAF Mutation	exon 15	3 (2.9%)
*n* = 3 (2.9%)		
PIK3CA	exon 9	3 (2.9%)
*n* = 7 (6.7%)	exon 20	4 (3.8%)
TP53	exon 4	5 (4.8%)
*n* = 78 (75%)	exon 5	23 (22.1%)
	exon 6	4 (3.8%)
	exon 7	19 (18.2%)
	exon 8	18 (17.3%)
	exon 9	3 (2.8%)
	exon 10	6 (5.7%)
RAS Wild Type	No	52 (50%)
	Yes	52 (50%)
RAS BRAF Wild Type	No	55 (52.9%)
	Yes	49 (47.1%)
All Wild Type	No	84 (80.8%)
	Yes	20 (19.2%)

ECOG PS, Eastern Cooperative Oncology Group Performance Status; FOLFOX, Folinic acid, Fluorouracil, and Oxaliplatin; CAPOX, Capecitabine and Oxaliplatin; FOLFIRI, Folinic acid, Fluorouracil, and Irinotecan; CAPEIRI, Capecitabine and Irinotecan; FUFOL, Folinic acid and Fluorouracil.

**Table 2:** Toxicity profile.

Toxicity Profile	Type	Value
Hematologic	Anemia	2 (2.70%)
	Neutropenia	3 (4.05%)
	Platelet decrease	0 (0.00%)
Nonhematologic	Liver	32 (43.20%)
	Cardiac	0 (0.00%)
	Neurologic	18 (24.30%)
	Digestive	10 (13.50%)
	Fatigue	25 (33.70%)
	Insomnia	3 (4.05%)
	Allergic reaction	2 (2.70%)
	Other	5 (6.80%)

**Table 3:** Important categorical variables.

Variables	Score
Curable disease	0.080979
Treatment setting	0.060790
Mucinous adenocarcinoma	0.044297
T4a	0.039078
Oligometastatic disease	0.031902
Absence of biologic treatment	0.031272
M1c	0.030110
Smoking	0.030024
KRAS	0.028041

T4a, infiltration of the serosa; M1c, spread to distant parts of the peritoneum (the abdominal cavity lining).

**Table 4:** Important numerical variables.

Variables	Score
WBC	0.128490
LDH	0.101582
ALP	0.090471
ASAT	0.069813
ANC	0.068928
GGT	0.061723
PLT	0.058054
Creatinine	0.049182
Dose reduction	0.045479
BUN	0.044141

WBC, white blood cells; LDH, lactate dehydrogenase; ALP, alkaline phosphatase; ASAT, aspartate transaminase; ANC, absolute neutrophil count; GGT, gamma-glutamyl transferase; PLT, platelets; BUN, blood urea nitrogen.

## Data Availability

The original contributions presented in the study are included in the article, further inquiries can be directed to the corresponding author.
